# Effects of Curcumin on the treatment of oral lichen planus symptoms: a systematic review and meta-analysis study

**DOI:** 10.1186/s12903-024-03873-y

**Published:** 2024-01-17

**Authors:** Hanie Moayeri, Abdolhalim Rajabi, Masoud Mohammadi, Sudabeh Bagheri Moghaddam

**Affiliations:** 1 Golestan University of Medical Sciences, Gorgan, Iran; 2 Health Management and Social Development Research Center, Department of Biostatistics and Epidemiology, Faculty of Health, Golestan University of Medical Sciences, Gorgan, Iran; 3 Golestan Research Center of Gastroenterology and Hepatology, Golestan University of Medical Sciences, Gorgan, Iran; 4 Maxillofacial Medicine, Dental Research Center, Department of Oral and Maxillofacial Medicine, Golestan University of Medical Sciences, Gorgan, Iran

**Keywords:** Mouth diseases, Autoimmune diseases, Oral Lichen Planus, Curcumin, Systematic review

## Abstract

**Background and objectives:**

Oral lichen planus (OLP) is a relatively common chronic T-cell–mediated disease that can cause significant pain, particularly in its erosive or ulcerative forms. This study aimed to examine the therapeutic impact of curcumin on symptoms of OLP.

**Materials and methods:**

This meta-analysis was performed according to the PRISMA guidelines. All related English documents indexed in electronic databases (including PubMed, Web of Science, Scopus, Embase, Wiley, Cochrane, and ProQuest databases [updated to August 15, 2023]) were retrieved. Data were double-extracted into a predefined worksheet, and quality analysis was performed using the Joanna Briggs Institute (JBI) scale. We carried out meta-analyses, and the random effects model was used to estimate the differences in erythema, lesion size, and pain between the curcumin control groups.

**Results:**

The search identified 289 studies, of which 10 were found to meet the inclusion criteria. The overall findings of the meta-analysis revealed that curcumin did not have a significant effect on erythema of OLP (standardized mean difference [SMD] = -0.14; 95% CI, -0.68 to 0.40; *P* = 0.61; I^2^ = 57.50%), lesion size of OLP (SMD = -0.15; 95% CI, -0.45 to 0.15; *P* = 0.33; I^2^ = 28.42%), and pain of OLP (SMD = -0.38; 95% CI, -0.97 to 0.22; *P* = 0.22; I^2^ = 86.60%). However, subgroup analysis based on treatment duration indicated that 2-week treatment duration was significantly associated with a reduction in OLP pain (*n* = 3; SMD = -1.21; 95% CI, -2.19 to -0.23; *P* = 0.01).

**Conclusions:**

Curcumin had no significant effect on erythema, lesion size, and pain of OLP compared to the control groups. However, subgroup analysis revealed that curcumin was more effective in reducing pain in non-randomized trials and in trials with a treatment duration of 2 weeks.

**Supplementary Information:**

The online version contains supplementary material available at 10.1186/s12903-024-03873-y.

## Introduction

Oral lichen planus (OLP) is a chronic inflammatory disease with lesions of varying severity and appearance. Its prevalence is about 0.5–2% [1]. The highest incidence of the disease is in the middle-aged population, with a female predominance with a ratio of approximately 2:1 [1, 2]. Oral lichen planus is a relatively common disease that occurs in the stratified squamous epithelium [3]. Oral lichen planus is important because it is a chronic disease that may affect the patient for years. It is associated with pain and burning sensation and has the potential to become malignant if not treated. There is no definitive cure for it [4]. The most common site of involvement is the buccal mucosa, along with the anterior two-thirds of the tongue, attached gingiva, labial mucosa, and vermilion of the lower lip [5]. The characteristic sign of OLP is “Wickham striae,” which appears as fine white lines with lace-like patterns [6]. There are various types of OLP, including reticular, erosive, bullous, and atrophic types [1]. The main goal of OLP treatment is to reduce painful symptoms and oral lesions, as well as to prevent lesions from turning into malignancy. The erosive and atrophic forms of OLP are more likely to turn into malignancy [7].

A common treatment for OLP is corticosteroids. Relapses may occur when steroids are discontinued. Therefore, the patients have to use medications for a long time [4]. Topical immunosuppressive drugs (such as cyclosporine, tacrolimus, pimecrolimus, and vitamin A metabolites) can be used to treat OLP, but due to their side effects, efforts are being made to consider an effective herbal medicine as a treatment method [8]. Systemic side effects of oral corticosteroids include immune system suppression, sleep disorders, and bone demineralization [9]. The long-term use of topical corticosteroids can cause secondary candidiasis, mucosal atrophy, taste changes, and burning sensation [10].

Researchers are looking for an alternative natural or herbal drug that can be used as monotherapy or in combination with first-line drugs to treat OLP. Curcumin is a non-toxic natural product that is used in various oral diseases, such as leukoplakia, oral submucosal fibrosis, and oral mucosal lesions caused by chemotherapy [11]. Curcumin has anti-inflammatory, antioxidant, antimicrobial, anticarcinogenic, antimutagenic, and antiproliferative properties and protects nervous and immune systems, which has been proven in many articles so far [6, 12–16]. It can eliminate reactive oxygen species, such as hydroxyl radicals and amine superoxide. Its antifungal properties prevent candidiasis, which is one of the common side effects of corticosteroids [17]. It is safe at very high doses [18].

In this regard, some studies suggest that curcumin may affect the treatment of OLP [1, 4, 19–22]. However, some other studies suggest that further investigations are needed to determine the effect of curcumin on OLP [6–8, 18, 23, 24]. Therefore, considering the importance of evidence-based decision-making and the need to infer from research in this regard, this systematic review and meta-analysis study examined the effect of curcumin on the treatment of OLP.

## Methods

This study was performed according to the PRISMA (Preferred Reporting Items for Systematic Reviews and Meta-Analyses) guidelines for systematic reviews in accordance with best practice guidelines [25].

The review protocol was registered in the PROSPERO (International Prospective Register of Systematic Reviews) database hosted by the National Institute for Health Research, University of York, Centre for Reviews and Dissemination (code: CRD42022323935).

### PICO question

The PICO strategy was developed in order to perform an accurate study retrieval and evaluation. Based on this, problem was OLP in adult patients (P); intervention studied was curcumin therapy (I); comparison was other kind of treatment or placebo (C) and main outcomes were symptoms of OLP (erythema, lesion size, and pain) (O).

### Search strategy

On 15 August 2023, we conducted a second search in the Google Scholar search engine and PubMed, Web of Science, Scopus, Embase, Wiley, Cochrane, and ProQuest databases. In this study, all the studies in English that were in the mentioned index were included in the study. A 3-step process was performed to determine the search keywords and design the search syntax. In the first stage, after PICO analysis, the concepts needed for the search were extracted according to the research topic. In addition, synonyms, abbreviations, related terms, UK/US spellings, singular/plural forms of words, and thesaurus terms (where available) were extracted and embedded in the search syntax to achieve maximum comprehensiveness in retrieving concepts. MeSH and Emtree terms were used to complete keywords and perform thematic searches on databases that have these tools. In the second stage, by conducting a preliminary search and analyzing the keywords of related and main articles, the vocabulary was completed and enriched. Therefore, the baseline syntax was (curcumin* OR turmeric) AND (lichen planus). Complete search strategies are presented in Appendix 1 for each database with their respective hits. In the third stage, the manual search of reference lists of all included studies and relevant systematic reviews was screened for any potentially eligible studies. Citation tracking was also performed for all the included articles.

### Data extraction

Two investigators (H.M. and M.M.) independently extracted the original data. Any disagreement was resolved by discussion. If a consensus was not reached, the results were reviewed by a third investigator (S.B.). The extracted data consisted of the following items: first author name, year of publication, country, type of study, sample size, study population, duration of treatment, type of outcome, type of intervention, and measure of effects and standard error.

To be included in the selection, studies had to meet the following criteria: being published in English, measuring one or more indicators of OLP symptoms as outcomes, providing sufficient quantitative data, and being interventional studies (randomized controlled trials or clinical trials). Studies that lacked a full-text publication in English were excluded, as well as reviews, observational studies, case reports, and comments.

### Assessment of risk of bias

Two independent investigators (A.R. and H.M.) assessed the quality of the included studies using the 13-point Joanna Briggs Institute (JBI) [26] scale for randomized controlled trials, with guidelines for assessing the risk of bias of randomized controlled trials (Appendix 2). All assessed studies were included in the analysis.

### Statistical analysis

The outcome measures of interest were erythema, lesion size, and pain. All were continuously measured. The standardized mean difference (SMD) for each outcome within each study was obtained by dividing the mean difference between children with curcumin and controls by the pooled SD, using the method described by Cohen [27] and converted effects to Hedges g [28]. Therefore, summary effect sizes and corresponding 95% CI were calculated using random effects models [29] for each outcome, using the method described by Dersimonian and Laird [30]. Heterogeneity was formally assessed using a chi-square test, with the significance level set at 0.10 [31]. The I^2^ index was used to indicate the percentage of total variation across studies attributable to heterogeneity rather than sampling error. We conducted a subgroup analysis based on the type of study, duration of treatment, and quality of studies. Statistical analyses were performed using Stata version 16 (StataCorp LLC, College Station, Texas, USA). P values less than 0.05 were considered statistically significant.

## Results

### Search results

**Fig. 1:**
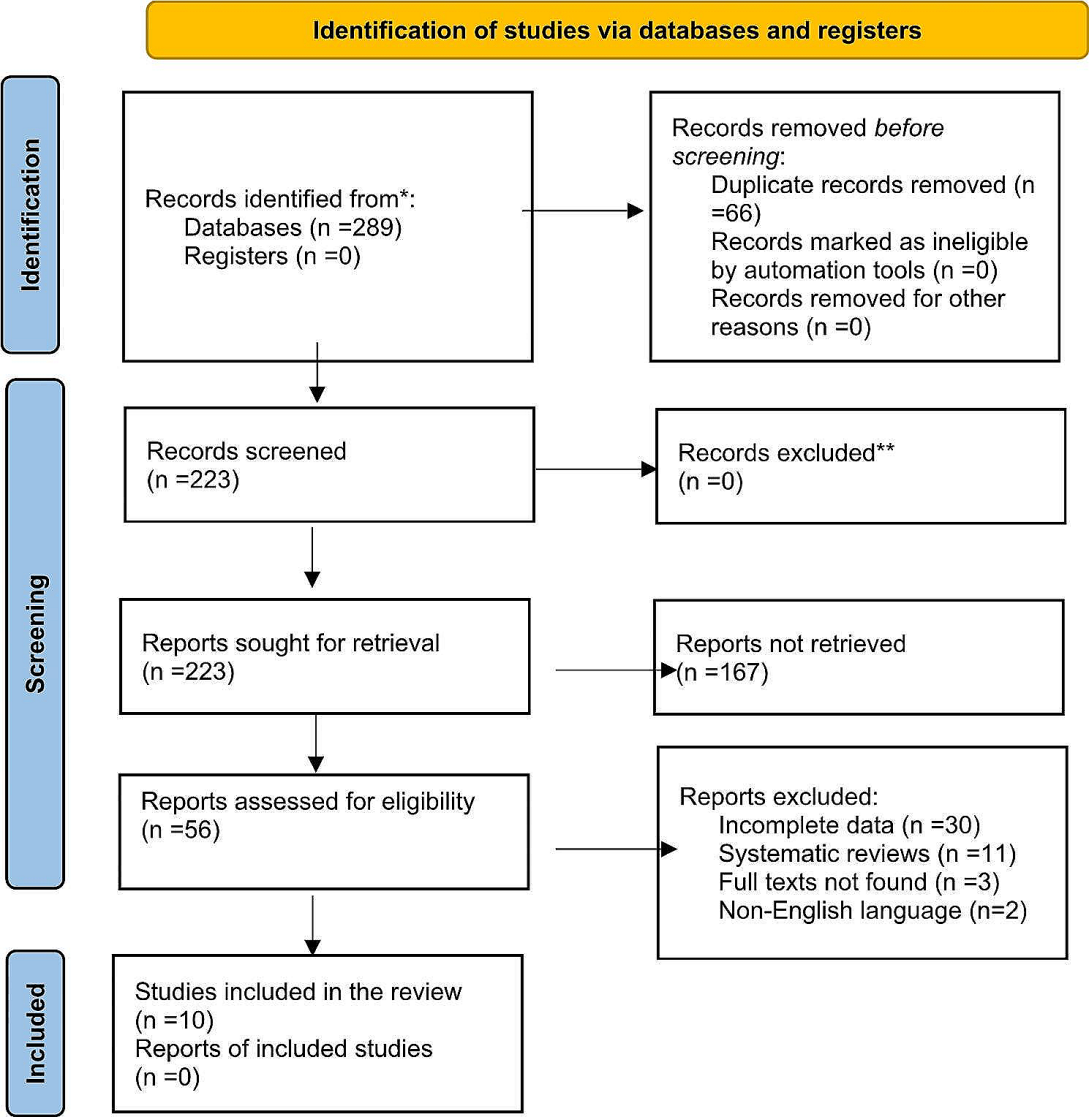
PRISMA flow diagram

A total of 289 studies were produced from the initial search. After removing duplicates, 223 abstracts were reviewed, with 56 selected for full-text review. No additional papers were identified through a bibliographic review. Overall, 10 articles were included in our meta-analysis (Fig. 1).

Ten studies, comprising 355 total patients, were selected for the final analysis. There were 8 randomized controlled trial studies and 2 non-randomized controlled trials. All 3 study outcomes were investigated in 2 studies, 2 desired outcomes in 5 studies, and only 1 desired outcome in the other 2 studies. Thus, there were 4 measures of effect indices for erythema, 7 for lesion size, and 9 for pain. The majority of the studies were conducted in Iran and India, with only one study from the USA. The follow-up duration for the erythema outcome ranged from two to 12 weeks, with most studies (five studies) reporting up to two weeks, one study reporting up to four weeks, and one study reporting up to 12 weeks. For the lesion size and pain outcomes, the follow-up duration was mostly four weeks, as reported by four studies. All the included studies used valid instruments to measure the outcomes (Table 1).

**Table 1:** Characteristics of the included studies in meta-analysis

First author, citation	Country	Type of study	Population	Duration of treatment	Type of outcome	Tools for assessing outcomes	Type of intervention	Type of intervention in the control group
Bakhshi et al., 2020 [17]	Iran	RCT	Patients with symptomatic (erosive and ulcerative) OLP who complained of pain and/or burning sensation participated in this study.	4 weeks	Erythema	REU Score	1% nanocurcumin gel	Placebo
Kia et al., 2015 [22]	Iran	RCT	50 patients (36 women and 14 men) in the age range of 38 to 73 and a mean age of 50.66 years. The patients had clinical signs of OLP (atrophic and ulcerative forms).	4 weeks	Lesion size	Thongprasom Scale (graded from 1 to 5)	Topical curcumin	Corticosteroid
Kia et al., 2015 [22]	Iran	RCT	50 patients (36 women and 14 men) in the age range of 38 to 73 and a mean age of 50.66 years. The patients had clinical signs of OLP (atrophic and ulcerative forms).	4 weeks	Pain	VAS (graded from 0 to 10)	Topical curcumin	Triamcinolone
Nosratzehi et al., 2018 [4]	Iran	RCT	The inclusion criterion was biopsy-confirmed OLP in combination with a compatible clinical appearance.	12 weeks	Pain	Pain Index	Curcumin mucoadhesive paste	Corticosteroid
Nosratzehi et al., 2018 [4]	Iran	RCT	The inclusion criterion was biopsy-confirmed OLP in combination with a compatible clinical appearance.	12 weeks	Lesion size	Grading from 0–3 cm	Curcumin mucoadhesive paste	Corticosteroid
Keshari et al., 2015 [21]	India	RCT	27 patients with symptomatic (atrophic/erosive) OLP were selected.	2 weeks	Lesion size	Semi-quantitative Scale (ranging from 0 to 3)	Topical curcumin ointment	Corticosteroid
Keshari et al., 2015 [21]	India	RCT	27 patients with symptomatic (atrophic/erosive) OLP were selected.	2 weeks	Pain	NRS (ranging from 0 to 10)	Topical curcumin ointment	Corticosteroid
Keshari et al., 2015 [21]	India	RCT	27 patients with symptomatic (atrophic/erosive) OLP were selected.	2 weeks	Erythema	Semi-quantitative Scale (ranging from 0 to 3)	Topical curcumin ointment	Corticosteroid
Thomas et al., 2017 [32]	India	RCT	75 patients who were diagnosed clinically and histopathologically with erosive OLP.	12 weeks	Erythema	Modified Oral Mucositis Index	1% curcuminoids as a local application	Placebo
Thomas et al., 2017 [32]	India	RCT	75 patients who were diagnosed clinically and histopathologically with erosive OLP.	12 weeks	Pain	NRS (ranging from 0 to 10)	1% curcuminoids as a local application	Placebo
Amirchaghmaghi et al., 2016 [18]	Iran	RCT	Patients with clinical signs of erosive-atrophic OLP confirmed by clinical or histopathological examination	4 weeks	Lesion size	Thongprasom Scale (graded from 1 to 5)	Curcumin tablets at a dose of 2000 mg/day	Placebo
Amirchaghmaghi et al., 2016 [18]	Iran	RCT	Patients with clinical signs of erosive-atrophic OLP confirmed by clinical or histopathological examination	4 weeks	Pain	VAS (graded from 0 to 10)	Curcumin tablets at a dose of 2000 mg/day	Placebo
Chainani-Wu et al., 2012 [33]	USA	CT	Patients with symptomatic OLP presenting to the oral medicine clinic at the University of California, San Francisco	2 weeks	Lesion size	Change in ulceration	Tablets with 95% curcuminoids	Placebo
Chainani-Wu et al., 2012 [33]	USA	CT	Patients with symptomatic OLP presenting to the oral medicine clinic at the University of California, San Francisco	2 weeks	Pain	NRS (ranging from 0 to 10)	Tablets with 95% curcuminoids	Placebo
Chainani-Wu et al., 2012 [33]	USA	CT	Patients with symptomatic OLP presenting to the oral medicine clinic at the University of California, San Francisco	2 weeks	Erythema	Modified Oral Mucositis Index	Tablets with 95% curcuminoids	Placebo
Naik et al., 2020 [37]	India	CT	Patients were selected from OPD randomly, and symptomatic OLP were included.	2 weeks	Pain	VAS (graded from 0 to 10)	Curcumin paste	Placebo
Kia et al., 2020 [11]	Iran	RCT	Patients with erosive and atrophic forms of OLP referred to the Oral Medicine Department	4 weeks	Lesion size	Thongprasom Scale (graded from 1 to 5)	Oral nanocurcumin	Corticosteroid
Kia et al., 2020 [11]	Iran	RCT	Patients with erosive and atrophic forms of OLP referred to the Oral Medicine Department	4 weeks	Pain	VAS (graded from 0 to 10)	Oral nanocurcumin	Corticosteroid
Ghobadi et al., 2022 [42]	Iran	RCT	28 patients with OLP were selected.	4 weeks	Lesion size	Change in size (2 mm)	1 Sina curcumin capsule (80 mg)	Placebo
Ghobadi et al., 2022 [42]	Iran	RCT	28 patients with OLP were selected.	4 weeks	Pain	VAS (graded from 0 to 10)	1 Sina curcumin capsule (80 mg)	Placebo

Abbreviations: RCT, randomized clinical trial; CT, clinical trial; OLP, oral lichen planus; OPD, outpatient department; REU, Reticulation/Keratosis, Erythema, and Ulceration; VAS, Visual Analog Scale; NRS, Numeric Rating Scale

### Meta-analysis results

**Fig. 2:**
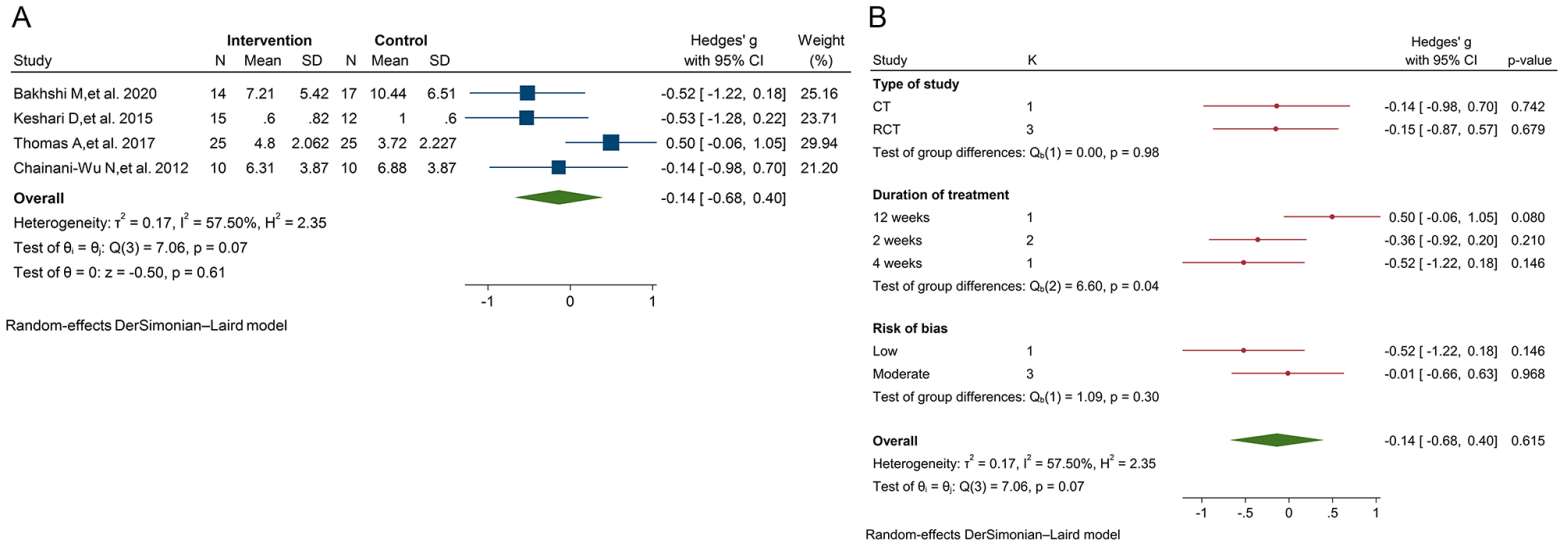
**A**. The forest plot of the random-effects meta-analysis of standardized mean difference (SMD) in erythema between the intervention group and control groups; **B**. The forest plot of the random-effects meta-analysis of standardized mean difference (SMD) in erythema between the intervention and control groups by subgroup analysis

**Fig. 3:**
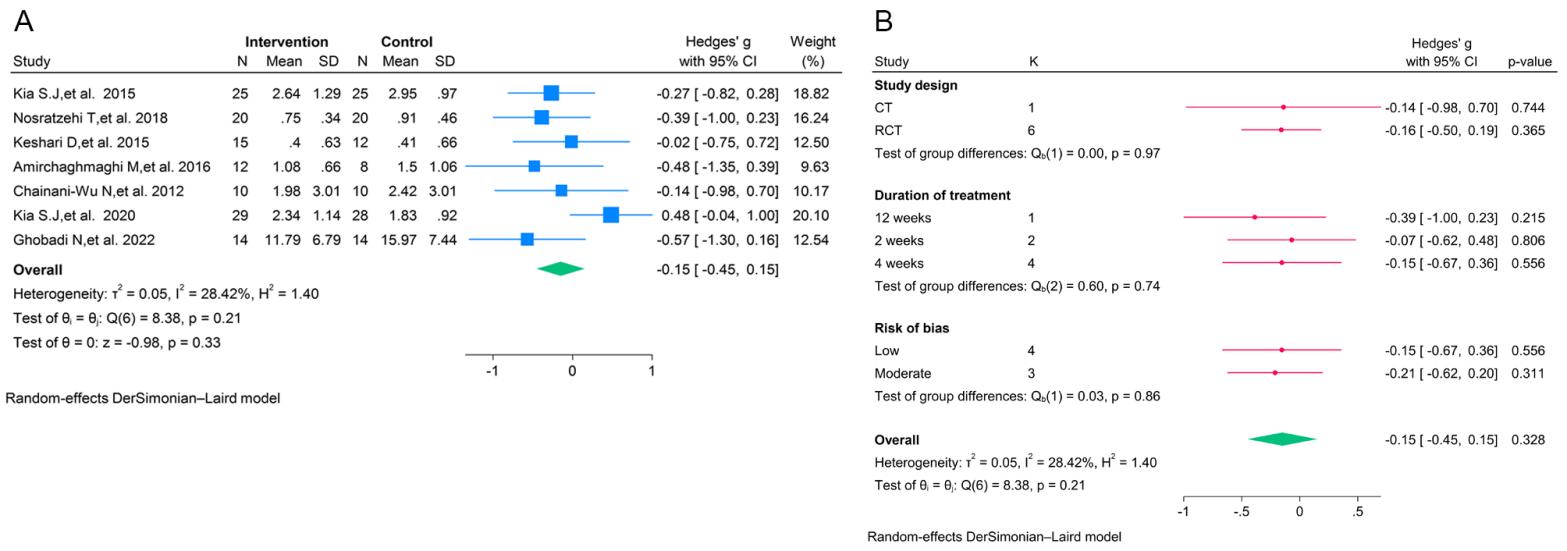
**A**. The forest plot of the random-effects meta-analysis of standardized mean difference (SMD) in lesion size between the intervention and control groups; **B**. The forest plot of the random-effects meta-analysis of standardized mean difference (SMD) in lesion size between the intervention and control groups by subgroup analysis

**Fig. 4:**
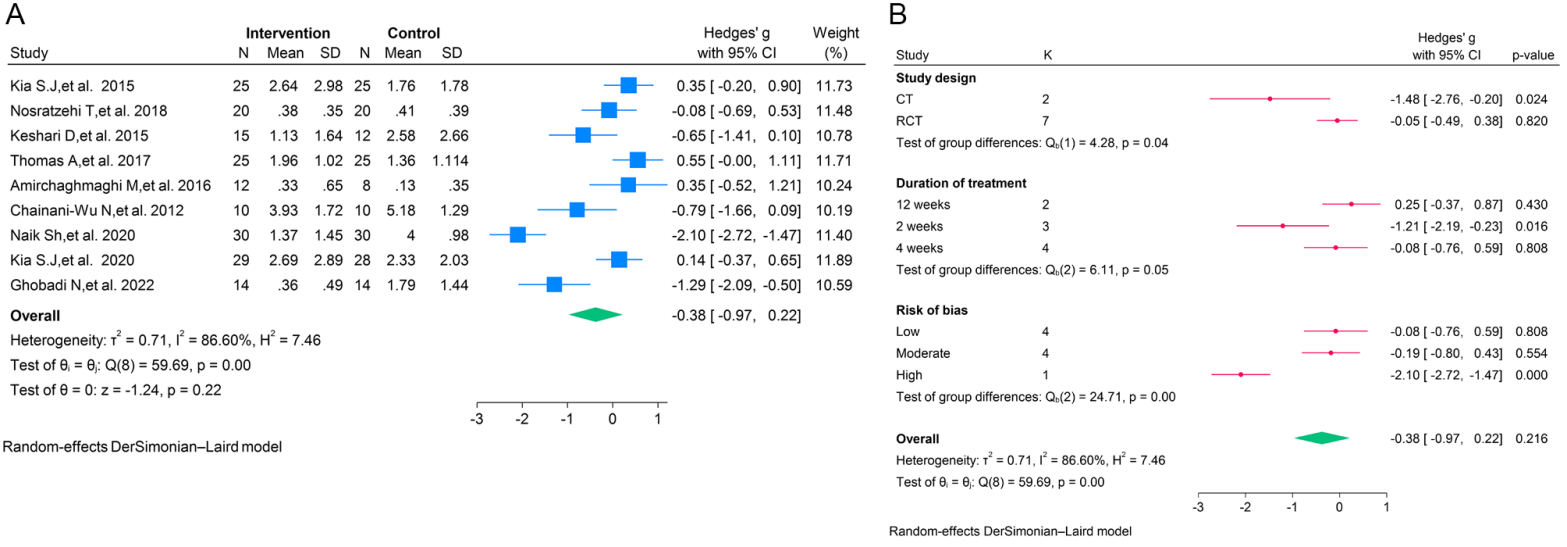
**A**. The forest plot of the random-effects meta-analysis of standardized mean difference (SMD) in pain between the intervention and control groups; **B**. The forest plot of the random-effects meta-analysis of standardized mean difference (SMD) in pain between the intervention and control groups by subgroup analysis

The results of the meta-analysis indicated that curcumin had no significant effect on the erythema of OLP (SMD = -0.14; 95% CI, -0.68 to 0.40; *P* = 0.61; I^2^ = 57.50%; Fig. 2A), lesion size of OLP (SMD = -0.15; 95% CI, -0.45 to 0.15; *P* = 0.33; I^2^ = 28.42%; Fig. 3A), and pain of OLP (SMD = -0.38; 95% CI, -0.97 to 0.22; *P* = 0.22; I^2^ = 86.60%; Fig. 4A).

Subgroup analysis based on the type of study, duration of treatment of curcumin, and quality of studies showed no significant decrease in the erythema and lesion size of OLP in the intervention groups relative to the control groups (Figs. 2B and 3B). However, subgroup analysis based on the type of study, duration of treatment with curcumin, and quality of studies revealed that the curcumin showed greater efficacy in controlling the pain of OLP in the non-randomized trials (*n* = 2; SMD = -1.48; 95% CI, -2.76 to -0.20; *P* = 0.02). In addition, the treatment duration of 2 weeks was significantly associated with the reduction of OLP pain (*n* = 3; SMD = -1.21; 95% CI, -2.19 to -0.23; *P* = 0.01; Fig. 4B).

### Publication bias

**Fig. 5:**
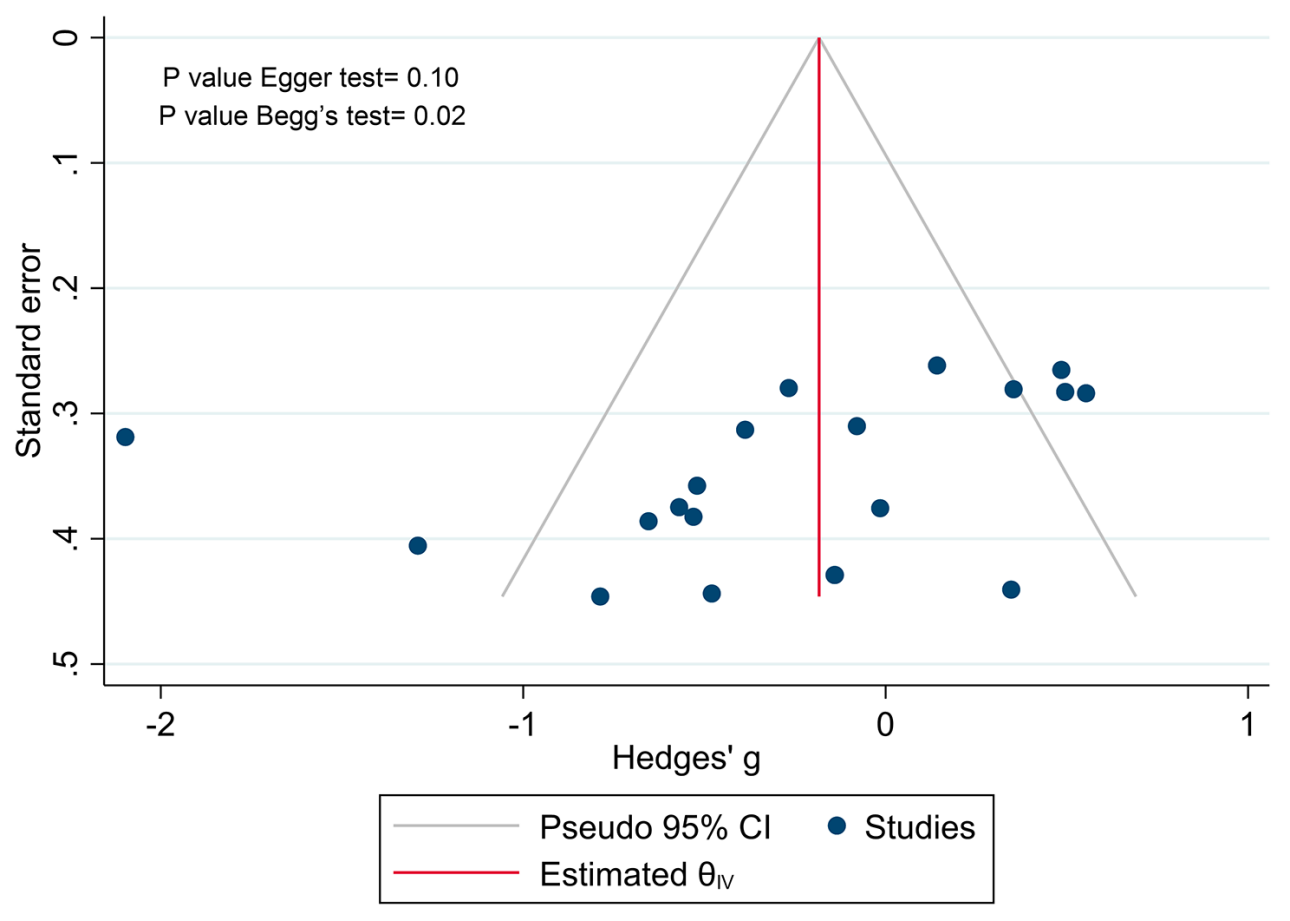
Funnel plot for included studies

We evaluated the publication bias in this study by using a funnel plot and two statistical tests: the Egger and the Begg’s tests. The funnel plot showed a symmetrical distribution of the effect sizes and their standard errors, indicating no significant publication bias. The Egger test also supported this finding, as it was not statistically significant (*p* = 0.1). However, the Begg’s test was statistically significant (*p* = 0.02), suggesting some degree of publication bias. To adjust for this possible bias, we applied the trim and fill method, which imputes the missing studies and recalculates the effect size. However, this method did not detect any missing studies, and the adjusted effect size was similar to the original one. This suggests that the publication bias was not severe enough to alter the results of this meta-analysis (Fig. 5).

### Risk of bias

Two authors (H.M. and A.R.) independently performed quality assessment for the included randomized controlled trials using the JBI scale for randomized controlled trial studies. Any discrepancies were discussed and agreed upon. The results are summarized in Table 2. Five studies had a low risk of bias, 4 had a moderate risk of bias, and 1 study had a high risk of bias.

**Table 2:** The bias risk assessment for the included randomized controlled trials using the Joanna Briggs institute scale for randomized controlled trials

First author, citation	Q1	Q2	Q3	Q4	Q5	Q6	Q7	Q8	Q9	Q10	Q11	Q12	Q13	Quality score	Risk of bias
Bakhshi et al., 2020 [17]	$\checkmark$	$\checkmark$	$\checkmark$	$\checkmark$	$\checkmark$	$\checkmark$	$\checkmark$	$\checkmark$	$\checkmark$	$\checkmark$	$\checkmark$	$\checkmark$	$\checkmark$	13	Low
Kia et al., 2015 [22]	$\checkmark$	$\checkmark$	$\checkmark$	$\checkmark$	$\checkmark$	$\checkmark$	$\checkmark$	$\checkmark$	$\checkmark$	$\checkmark$	$\checkmark$	$\checkmark$	$\checkmark$	13	Low
Nosratzehi et al., 2018 [4]	×	×	$\checkmark$	×	×	×	$\checkmark$	$\checkmark$	$\checkmark$	$\checkmark$	$\checkmark$	$\checkmark$	×	7	Moderate
Keshari et al., 2015 [21]	$\checkmark$	×	$\checkmark$	×	×	×	$\checkmark$	$\checkmark$	$\checkmark$	$\checkmark$	$\checkmark$	$\checkmark$	×	8	Moderate
Thomas et al., 2017 [32]	$\checkmark$	×	$\checkmark$	×	×	×	$\checkmark$	$\checkmark$	$\checkmark$	$\checkmark$	$\checkmark$	$\checkmark$	×	8	Moderate
Amirchaghmaghi et al., 2016 [18]	$\checkmark$	$\checkmark$	$\checkmark$	$\checkmark$	$\checkmark$	$\checkmark$	$\checkmark$	$\checkmark$	$\checkmark$	$\checkmark$	$\checkmark$	$\checkmark$	$\checkmark$	13	Low
Chainani-Wu et al., 2012 [33]	×	×	$\checkmark$	×	×	×	$\checkmark$	$\checkmark$	$\checkmark$	$\checkmark$	$\checkmark$	$\checkmark$	×	7	Moderate
Naik et al., 2020 [37]	×	×	$\checkmark$	×	×	×	$\checkmark$	$\checkmark$	×	$\checkmark$	$\checkmark$	$\checkmark$	×	6	High
Kia et al., 2020 [11]	$\checkmark$	$\checkmark$	$\checkmark$	$\checkmark$	$\checkmark$	$\checkmark$	$\checkmark$	$\checkmark$	$\checkmark$	$\checkmark$	$\checkmark$	$\checkmark$	$\checkmark$	13	Low
Ghobadi et al., 2022 [42]	$\checkmark$	$\checkmark$	$\checkmark$	$\checkmark$	×	×	$\checkmark$	$\checkmark$	$\checkmark$	$\checkmark$	$\checkmark$	$\checkmark$	×	10	Low

## Discussion

The aim of this study was to investigate the effect of all types of curcumin on the treatment of OLP. The total number of articles after removing duplicates was 223, of which 10 were included in the meta-analysis. Erythema, lesion size, pain, duration of treatment, and type of study were the variables of this study. In this study, curcumin was evaluated topically or systemically with or without corticosteroids.

In the present study, curcumin had no significant effect on erythema in OLP. Consistent with our study, in the studies by Bakhshi et al. [17], Keshari et al. [21], Thomas et al. [32], and Chainani-Wu et al. [33], there was no significant effect on erythema in patients with OLP. In the present study, curcumin had no statistically significant effect on the lesion size of patients with OLP. The 12-week treatment period, compared to the 2- and 4-week treatment periods, showed a significant effect in reducing the lesion size. In the studies by Kia et al. [11, 22], the lesion size was reduced in the intervention group, which is consistent with the current study. In the study by Nosratzehi et al. [4], there was no significant effect on the reduction of the lesion size after a 12-week follow-up period. In the study by Kapoor and Arora [34], after a 12-week follow-up period, a great and statistically significant decrease was seen in the size of the lesion.

Curcuminoid gel 1% is not as effective as triamcinolone acetonide 0.1% in reducing burning sensation and erythema and lesion changes [20, 32], while topical application of high-dose curcumin is as effective as topical application of corticosteroid [4, 22]. Therefore, the effectiveness of curcumin in the form of oral paste is greater and longer compared to its gel format, as the paste stays on the mucosa for a longer period of time. Although topical curcumin is effective in the treatment of OLP, it still cannot replace topical corticosteroids for many patients [9].

In a study [18], the use of curcumin in tablet form (systemic) did not show a significant difference between the intervention and control groups; in addition, according to another study [35], it can be stated that curcumin in capsule form is a suboptimal dose because it does not allow the drug to reach the lesions through direct contact. It was shown that oral curcumin could be effective in preventing the recurrence of OLP lesions after treatment and initial control; in addition, the dose of curcumin is more important than the duration of the treatment [11]. Studies [23, 33, 36] have shown that curcumin with a dose of 2000 mg/day did not show a significant difference between the intervention and control groups, while curcumin with a dose of 6000 mg/day showed a significant improvement; this shows that curcumin, like many drugs, may have a dose-dependent effect. Curcumin in the prescribed dose is safe and effective in controlling the signs and symptoms of OLP and can reduce the need for drugs such as corticosteroids [6].

The present study indicated that curcumin had no significant effect on the improvement of pain in patients with OLP after 4 and 12 weeks; however, the 2-week treatment period showed a significant effect on reducing the pain of patients. In the studies by Naik et al. [37] and Chainani-Wu et al. [33], pain in patients with OLP was reduced after 2 weeks. However, consistent with the current study, Nosratzehi et al. [4] and Thomas et al. [32] after a 12-week follow-up and Kia et al. [11, 22] and Amirchaghmaghi et al. [18] after a 4-week follow-up showed a weak and non-significant effect on pain improvement. Curcumin is used as a wound healing agent and has anti-inflammatory, antioxidant, analgesic, antiparasitic, and antimalarial effects. Its anti-inflammatory and healing properties have been mentioned in previous studies [4, 16, 38–41]. Therefore, it can be concluded that curcumin has acceptable anti-inflammatory and healing effects.

This review has some limitations. Overall, there was a small number of total patients included in the data, which made it infeasible to construct a funnel plot to assess the variation and publication bias in the studies. Also, the tools for measuring OLP symptoms were not the same in all studies, which can cause differences in the precision of measuring these symptoms in studies. As a result, it may affect the pooled result of the meta-analysis. In addition, the form of curcumin used in treating patients was not the same in all studies.

## Conclusions

Curcumin had no significant effect on erythema, lesion size, and pain of OLP compared to the control groups. However, subgroup analysis revealed that curcumin was more effective in reducing pain in non-randomized trials and in trials with a treatment duration of 2 weeks. The quality assessment of the included studies indicated that most of them had a low or moderate risk of bias, but some limitations were noted, such as a small sample size, lack of blinding, and heterogeneity among studies. Therefore, more high-quality randomized controlled trials are needed to confirm the findings of this meta-analysis and explore the optimal dose and duration of curcumin for OLP. Curcumin is a natural compound with anti-inflammatory, antioxidant, and immunomodulatory properties that may have potential benefits for various inflammatory diseases. However, its clinical efficacy and safety for OLP remain unclear and warrant further investigation.

### Electronic supplementary material

Below is the link to the electronic supplementary material.

Supplementary Material 1

Supplementary Material 2

## Data Availability

The datasets used and/or analyzed during the current study are available from the corresponding author on reasonable request.

## Abbreviations

SMD: Standardized mean difference
OLP: Oral lichen planus
PRISMA: Preferred Reporting Items for Systematic Reviews and Meta-Analyses
