# Supplementary material for: Effects of Curcumin on the treatment of oral lichen planus symptoms: a systematic review and meta-analysis study
Source: BMC Oral Health. 2024 Jan 17;24:104. doi: 10.1186/s12903-024-03873-y (PMC10795217; doi:10.1186/s12903-024-03873-y)
Supplement: Supplementary file 1 — Supplementary Material 1 [file 12903_2024_3873_MOESM1_ESM.docx]

**August 15 2023**

**Main syntax: (curcumin* OR turmeric) AND (Lichen Planus)**

| **Database** | **Formula** | **No. of Results** |
| --- | --- | --- |
| PubMed | (("Curcuma"[Mesh]) AND "Lichen Planus"[Mesh]) OR ((curcumin*[Title/Abstract] OR turmeric[Title/Abstract]) AND (Lichen Planus[Title/Abstract])) | 29 |
| Scopus | ( ( TITLE-ABS-KEY ( curcumin* ) ) OR ( TITLE-ABS-KEY ( turmeric ) ) ) AND ( TITLE-ABS-KEY ( lichen AND planus ) ) | 89 |
| Web of Science | TOPIC: (curcumin*  OR turmeric) AND TOPIC: (Lichen Planus)  Timespan: All years. Indexes: SCI-EXPANDED, SSCI, A&HCI, CPCI-S, CPCI-SSH, BKCI-S, BKCI-SSH, ESCI, CCR-EXPANDED, IC. | 41 |
| Embase | ((curcumin*:ti,ab,kw OR turmeric:ti,ab,kw) AND 'lichen planus':ti,ab,kw) OR (('curcumin'/exp OR 'turmeric'/exp AND 'lichen planus'/exp)) | 90 |
| ProQuest | abstract((curcumin* OR turmeric) AND (Lichen Planus)) OR subject((curcumin* OR turmeric) AND (Lichen Planus)) OR title((curcumin* OR turmeric) AND (Lichen Planus)) | 57 |
| OVID | ((curcumin* or turmeric) and Lichen Planus).ab. or ((curcumin* or turmeric) and Lichen Planus).at. OR ((curcumin* or turmeric) and Lichen Planus).kw. | 36 |
| Cochrane | ((curcumin* OR turmeric):ti,ab,kw AND (lichen planus):ti,ab,kw) OR ((MeSH descriptor: [Curcuma] explode all trees OR MeSH descriptor: [Curcumin] explode all trees)) | 26 |
| Wiley | "curcumin* OR turmeric" in Abstract and "Lichen Planus" in Abstract | 8 |
| Google Scholar ([Search English pages](https://scholar.google.com/scholar?lr=lang_en&q=(curcumin*+OR+turmeric)+AND+Lichen+Planus&hl=en&as_sdt=0,5)) | (curcumin OR turmeric) AND (Lichen Planus) | 214 |
| Number of results after deleting duplicated files | 582 | |
